# Variations in Acorn Characteristics Between Two Mediterranean *Quercus* Species and Their Hybrids Through Contrasting Environmental Gradients in Spain

**DOI:** 10.3390/plants14050718

**Published:** 2025-02-26

**Authors:** Santiago González-Carrera, Alfonso Escudero, Alejandro Fernández-Fuentes, Montserrat Martínez-Ortega, Sonia Mediavilla

**Affiliations:** 1 Department of Ecology, Faculty of Biology, University of Salamanca, 37071 Salamanca, Spain; santiagojosegc@usal.es (S.G.-C.); ecoescu@usal.es (A.E.); alejandrofuentes@usal.es (A.F.-F.); 2 Department of Botany and Plant Physiology, Faculty of Biology, University of Salamanca, 37071 Salamanca, Spain; mmo@usal.es; 3 Herbarium and Plant DNA Biobank, University of Salamanca, 37071 Salamanca, Spain

**Keywords:** acorn morphology, climatic gradients, fruit plasticity, hybridization, *Quercus* spp.

## Abstract

Oaks are characterized by high plasticity and intense interspecific gene flow due to natural hybridization. This generates a wide phenotypic spectrum, which creates taxonomic confusion within the genus. We compared the acorn traits across a temperature gradient in two types of Mediterranean *Quercus* (*Quercus faginea* Lam. and *Q. pyrenaica* Willd.) and their hybrids. Genetic groups were identified using amplified fragment length polymorphism (AFLPs) analysis. Acorns sampled from each of the three genetic groups were used for comparative purposes by means of 15 morphological characteristics. Eight of the traits showed discriminant value among the three groups. The acorn height tended to decrease with decreasing temperatures across the gradient, whereas the acorn width exhibited the opposite response. However, fruit traits allowed discrimination between the three groups, and the differences were consistent in the different zones. Both the number of acorns produced and the individual acorn size were larger for *Q. pyrenaica*. Hybrids showed intermediate traits between both parent species. Traditionally, the persistence of parental species in the absence of reproductive barriers has been explained by the lower fitness of the hybrids. Our results, however, do not reveal the presence of transgressive characteristics in the hybrids that could justify a lower competitive capacity.

## 1. Introduction

The leaf morphology has traditionally been one of the main aspects used in plant species delimitation [1,2,3]. However, the use of leaf traits as discriminants poses some problems, particularly for some genera, such as *Quercus*. Oak species are characterized by their high leaf plasticity in response to environmental changes [4,5,6], which generates strong variability in the leaf morphology among species, among populations of the same species, among individuals of a population, and even within the crown of a single specimen [7,8,9]. *Quercus* species are also known for their intense gene flow due to frequent phenomena of natural hybridization [10,11,12]. This generates a wide phenotypic spectrum, making identification difficult and producing taxonomic confusion within the genus. Both within-species plasticity and hybridization can lead to uncertainties when the taxonomical identification of oak individuals is based exclusively on the observation of morphological leaf traits [13,14,15]. For this reason, different authors have also proposed the use of reproductive characteristics, specifically the acorn morphology, for the classification of oak species and determination of hybridization, and numerous studies have indeed revealed their taxonomic utility in the study of the genus [16,17]. Since the characteristics of reproductive organs are key ecological traits that play a fundamental role in plant life cycles (affecting the rates of germination; the emergence, development, and survival of the seedlings; predation; and dispersal), their study can also provide key information to understand the patterns of natural regeneration and distribution in different species [18]. Additionally, knowing the pattern of variation in the fruit morphology and its relationship with environmental factors can help us to infer the consequences of current environmental changes for population persistence and the role of genetics and the environment in plant adaptation.

In this study, we set out to characterize the acorn morphology and production in two types of Mediterranean *Quercus* (*Quercus faginea* Lam. and *Q. pyrenaica* Willd.) and their hybrids, preliminarily identified from molecular markers, and to analyze their changes across a climatic gradient. These two species are among the most widespread oaks in the Iberian Peninsula. Both are deciduous and coexist in many places. Previous studies of these two closely related species have addressed some aspects related to their leaf morphology and physiology, as well as their ecological requirements, highlighting significant differences between them [6,19]. However, we do not know of any study that has compared the morphological characteristics of the acorns of both species and their hybrids, although they share numerous areas with high levels of hybridization [20]. Although between-population variability in the acorn morphology has been observed in different *Quercus* species over a broad range of conditions [21,22,23], we do not know of studies addressing the plasticity in the fruits of *Q. faginea* and *Q. pyrenaica* in response to changes across environmental gradients, despite the importance of trait plasticity for the persistence of both species under the new expected climatic conditions. Mediterranean deciduous oaks are known for their extreme interannual variability in fruit production [24]. In the two studied species, acorn production may be almost nil for years, so fruit traits can only be adequately characterized during mast years. During the past year (2024), an extremely large amount of acorn production has been recorded in our study region, with strong synchronization between both species and different populations, giving us the opportunity to compare the acorn characteristics of the different genetic groups both within the same geographical area and across a climatic gradient.

In a previous study of the leaf morphologies of the same species and their hybrids [20], we observed that, although practically all of the morphological traits studied differed significantly between the two species, the hybrids showed, in general, leaf traits that were more similar to those of one of the parental species (*Q. faginea*), so that the characteristics related to the leaf morphology did not seem to offer much discriminant value in our study complex. Among the three genetic groups (*Q. faginea*, *Q. pyrenaica*, and hybrids), *Q. faginea* exhibited the strongest responses in its leaf traits to temperature changes across a geographic gradient. Since greater phenotypic plasticity has been considered important to allow plants to respond successfully to changing environmental conditions [25,26], the greater plasticity of *Q. faginea* could confer an advantage to this species compared to *Q. pyrenaica* and to the hybrids in the face of the changing climatic conditions predicted.

In this work, firstly, we propose to study the acorn characteristics of the two species and their hybrids and to detect the characteristics that allow discrimination among the three genotypes. We intend to verify whether, unlike the leaf morphology, the fruit morphology does offer a criterion for the identification of hybrids. Secondly, we analyze the variability in the fruit traits in response to changes across a climatic gradient and check whether there are differences in plasticity among traits and among the three genetic groups (the two species and their hybrids). Thirdly, we check whether the possible differences among the three genetic groups are similar in different areas distributed across a climatic gradient. Given the crucial influence of the size and other acorn traits in the regeneration of oak forests, analyzing the fruit responses to climatic gradients is especially important to formulate predictions about the future evolution of forest masses in one of the climates (Mediterranean) and one of the areas (the Iberian Peninsula) considered most vulnerable in a climate change scenario [27,28].

## 2. Results

### 2.1. Effects of Taxa on Fruit Trait Variability

**Figure 1:**
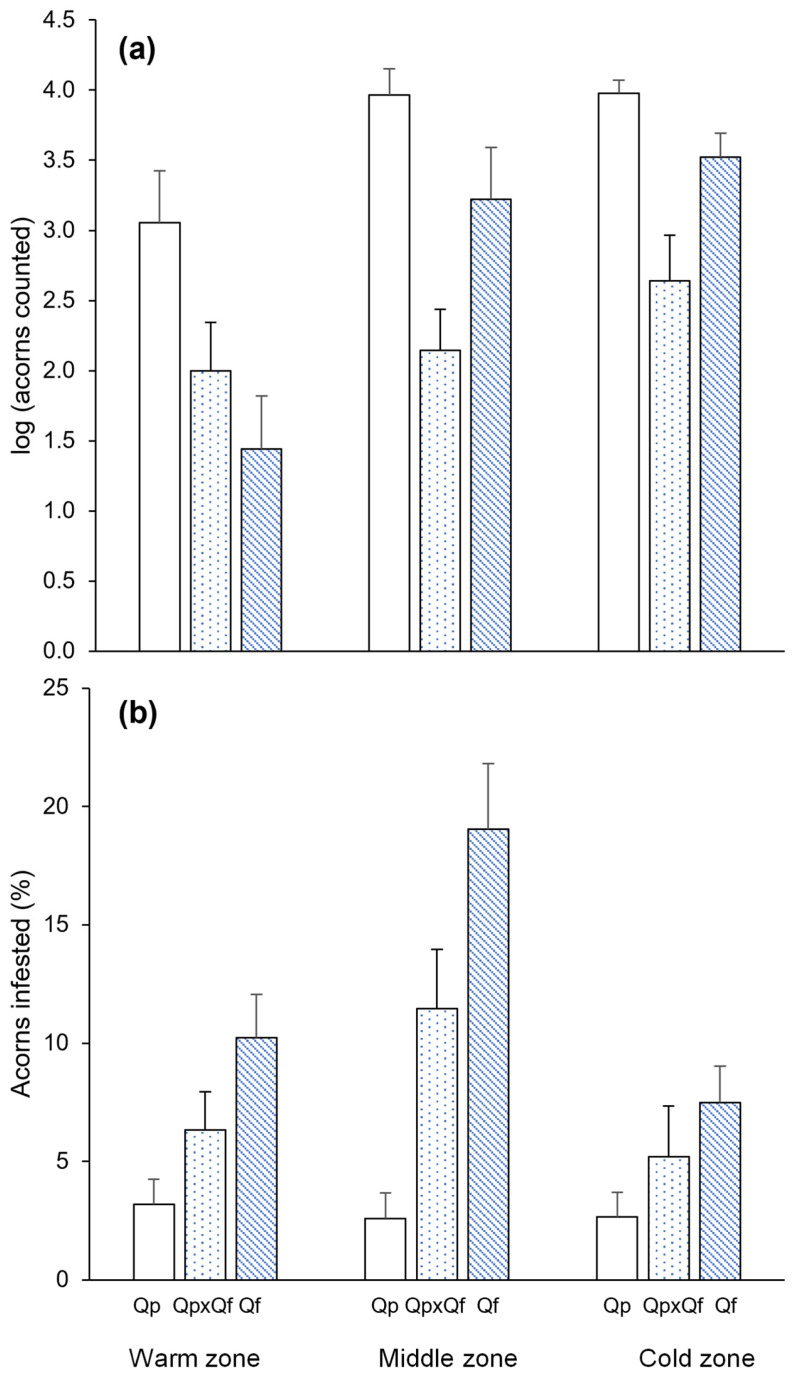
Mean (+SE) of (**a**) acorn production (30 s counts in visual surveys, log-transformed) and (**b**) the percentage of acorns infested by insect larvae for the different genetic groups (*Quercus pyrenaica*, Qp; hybrids, Qp x Qf; *Q. faginea*, Qf) in the three climatic zones.

Visual counts indicated significantly higher acorn production in *Q. pyrenaica* (around 50% more acorns counted than for *Q. faginea*) in the three study areas (Figure 1a). Among the other two groups, acorn production was lower in the hybrids in the cold and middle zones, and it was only lower in *Q. faginea* than in the hybrids in the warm zone (Figure 1a). *Q. pyrenaica* was also the group with the lowest percentage of acorns predated by insect larvae, regardless of the study area, while *Q. faginea* showed the highest percentage of acorn predation, with the hybrids exhibiting intermediate values (Figure 1b).

**Figure 2:**
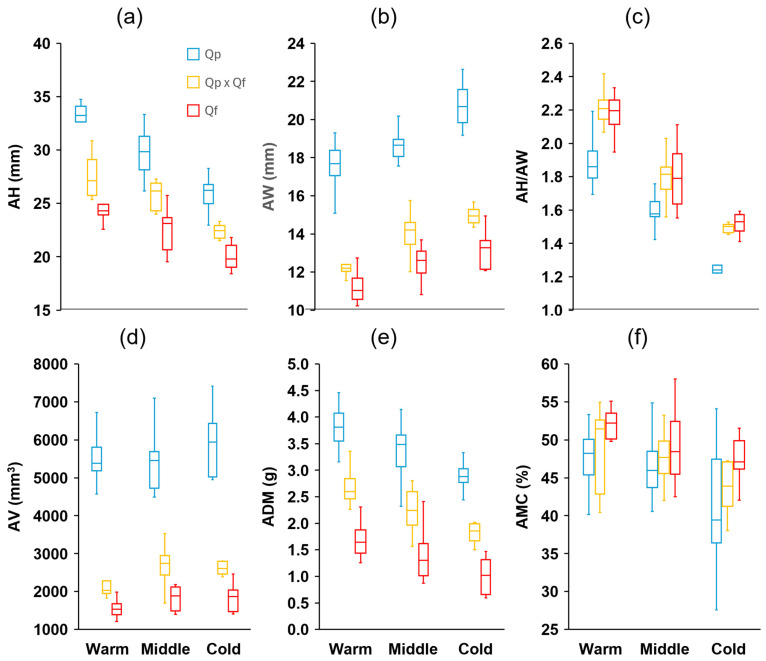
Boxplot diagrams of (**a**) height (AH), (**b**) width (AW), (**c**) AH/AW ratio, (**d**) volume (AV), (**e**) dry mass (ADM), and (**f**) moisture content (AMC) of acorns from the different genetic groups (*Quercus pyrenaica*, Qp; hybrids, Qp x Qf; *Q. faginea*, Qf) in the three climatic zones. The box in each box plot shows the median and the lower and upper quartiles, and the whiskers show the range of variation.

**Figure 3:**
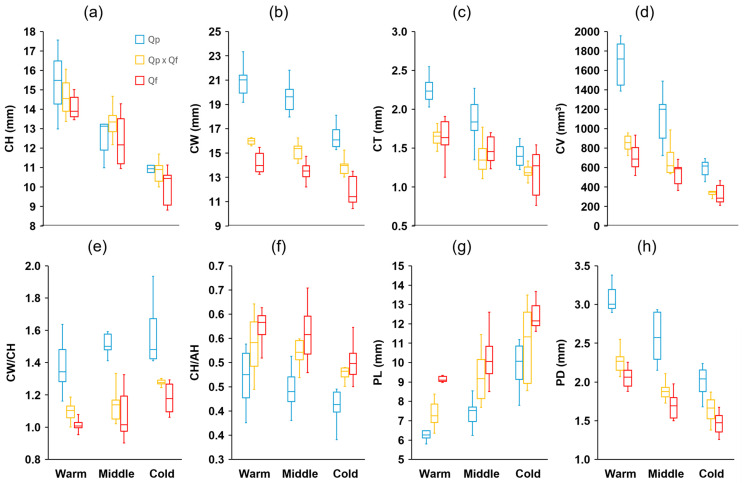
Boxplot diagrams of (**a**) height (CH), (**b**) width (CW), (**c**) thickness (CT), (**d**) volume (CV), (**e**) CW/CH ratio, (**f**) CH/AH ratio of the cupules, and (**g**) length (PL) and (**h**) diameter (PD) of the peduncles of acorns from the different genetic groups (*Quercus pyrenaica*, Qp; hybrids, Qp x Qf; *Q. faginea*, Qf) in the three climatic zones. The box in each box plot shows the median and the lower and upper quartiles, and the whiskers show the range of variation.

Measurements of 15 characteristics were taken from a total of 900 acorns (100 per site, 10 randomly chosen per tree). These characteristics’ definitions and units are summarized in Table 1 (see Section 4). There were significant differences among the genetic groups in all fruit traits analyzed (Table S1). Eight traits showed discriminant value between the three groups (post hoc Tukey–Kramer HSD test), with *Q. pyrenaica* presenting larger acorns (length, width, volume, and mass), wider cupules, and shorter but thicker peduncles, while the opposite was observed in *Q. faginea*, with intermediate values in general for the hybrids (Figure 2 and Figure 3). The mean acorn dry mass in *Q. pyrenaica* amounted to more than twice that of *Q. faginea*. For the remaining traits, differences were evident between the two species, with lower AH/AW (Figure 2) and CH/AH but higher CW, CT, CV, and CW/CH (Figure 3) in *Q. pyrenaica* than in *Q. faginea*. For most of these traits, the hybrids showed similar values to *Q. faginea*. The height and width of acorns and cupules, as well as the peduncle characteristics, were the traits that showed the greatest consistency among individuals from the same population, with CVs always lower than 10–15% (Table S2), while the acorn mass and volume and cupule volume were the characteristics with the highest variability in general (more than 20% in some cases). Contrary to our expectations, the CVs were not generally greater for the hybrids than for the parental species.

**Figure 4:**
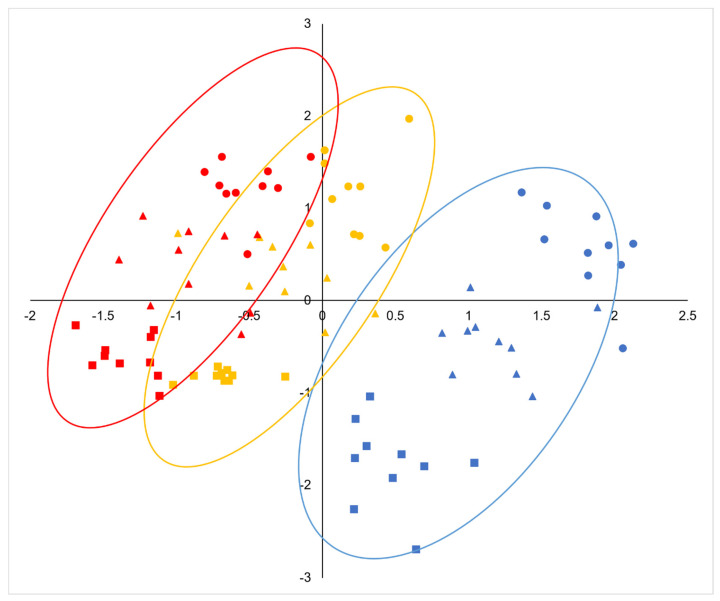
Principal component analysis (PCA) based on the morphological and anatomical acorn traits (genetic groups are labeled with different colors: *Quercus pyrenaica*, blue, hybrids, orange, *Q. faginea*, red; climatic zones are labeled with different symbols: warm, circle, intermediate, triangle, cold, square).

**Figure 5:**
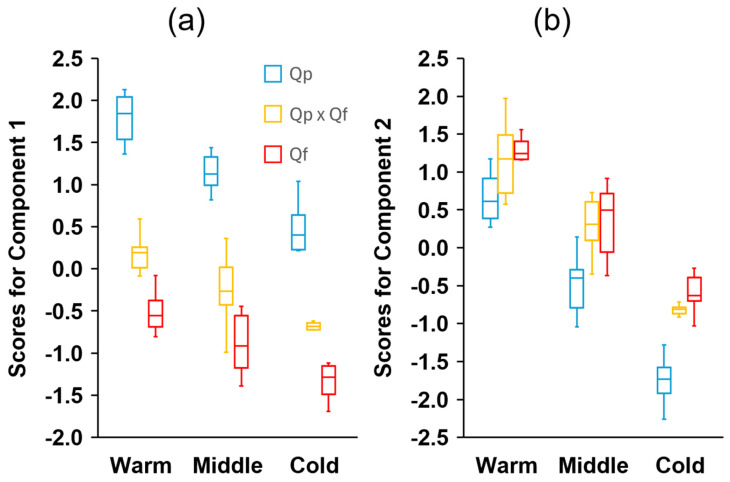
Boxplot diagrams depicting the distribution of the individual scores for (**a**) the first and (**b**) the second principal components of the different genetic groups (*Quercus pyrenaica*, Qp; hybrids, Qp x Qf; *Q. faginea*, Qf) in the three climatic zones. The box in each box plot shows the median and the lower and upper quartiles, and the whiskers show the range of variation.

There were significant correlations between most of the fruit dimensions measured, being especially strong between the width of the acorn and cupule and their respective volumes, the height and mass of the acorns, the mass and moisture content, and the size of the acorn and cupule and the diameter of the peduncle (Table S3). Larger acorns have larger and thicker cupules, but less covering (lower CH/AH ratio) and shorter but thicker peduncles (Table S3). Based on the selected fruit traits, the PCA revealed that the first and second principal components explained 82.2% of the total variance, with the majority, 54.8%, corresponding to PC1 (Table 2). The variables that most contributed to the first principal component were CW, CV, the acorn fresh and dry mass, the acorn height, and PD. The ratio AH/AW and CH for the positive side and CW/CH and AW for the negative side exhibited the strongest correlations with PC2 (Table 2). The first component clearly discriminated *Q. pyrenaica* (distributed on the right side of the diagram) from *Q. faginea* (Figure 4). Hybrids tended to occupy the central part of the axis, exhibiting intermediate mean individual scores (Figure 5), although they were somewhat closer to *Q. faginea* individuals. The second principal component clearly discriminated the three zones across the gradient, with the warmest at the top, the coldest at the bottom, and the intermediate zone occupying positions between the two (Figure 4). The three genetic groups exhibited similar scores with respect to PC2 but they tended to be more negative for *Q. pyrenaica* in the coldest sites (Figure 5).

### 2.2. Effects of Environmental Gradients on Fruit Trait Variability

There were differences between the areas across the climatic gradient in all traits analyzed (Table S1). In 10 of these traits, the differences were significant between the three zones (post hoc Tukey–Kramer HSD test). For the three genetic groups, the acorn height tended to decrease with the decrease in temperature across the gradient, whereas the acorn width exhibited the opposite response (Figure 2). Accordingly, the ratio AH/AW decreased significantly as the mean temperature decreased. The same trend was also apparent for the mean dry mass (ADM, Figure 2), although the mean acorn volume did not change within a single group across the gradient. The three groups tended to produce shorter and narrower cupules, with a smaller thickness (CT) and volume (CV), as well as longer peduncles, but with a smaller diameter (Figure 3) as the temperatures decreased between the zones through the gradient. In the remaining traits, the differences among the zones were less apparent. The ratio CH/AH showed a slight decrease (smaller proportion of the acorn covered by the cupule) across the gradient (Figure 3). Finally, the moisture content (AMC) tended to decrease with decreasing temperatures, although the differences were only evident for the coldest sites (Figure 2).

The combinations of traits present in the different climatic zones showed a consistent change, according to the principal component analysis (Figure 4). For the three genetic groups, the mean individual scores for PC1 and PC2 decreased consistently with decreasing temperatures (Figure 5).

Among the traits that responded to environmental changes, PL, AH/AW, the acorn weight, and CT, and especially CV, were the most variable between the zones within a single group, as indicated by their plasticity indices ranging between 0.20 and 0.40 in most cases (Table 3). *Q. pyrenaica* tended to exhibit the highest variability for most cupule traits, whereas *Q. faginea* showed high plasticity in the individual acorn mass (more than 0.40) in response to climatic changes between the sites (Table 3).

## 3. Discussion

Given the strong variability that characterizes the leaves of the species of the genus *Quercus*, several authors have proposed including the use of fruit characteristics for the classification of oak species and determination of hybridization [16,17]. Other authors, however, do not consider the fruit morphology to be a reliable discriminant between oak species and their hybrids, either because their studies did not reveal large differences between genetic groups or because of the enormous variation found in these traits within and between populations and individuals [29,30,31]. In the specific case of our two *Quercus* species, the results revealed that fruits allow the discrimination of the two studied species, showing significant differences in all features considered. In addition, the fruits showed discriminant value for the characterization of hybrids, with 8 of the 15 traits analyzed allowing them to be significantly distinguished from the two parental species and with intermediate values between both. Since these results have been obtained during a single year of sampling, they must be interpreted with caution. The masting behavior in our oak species makes it extremely difficult to obtain comparable data on the fruit morphology for different species under the same conditions. It is known that some fruit traits may be affected by environmental conditions, which implies that different species can only be compared under similar circumstances. The masting episode of 2024 was an opportunity to obtain data for the three genetic groups during the same time period and in the same sites. In fact, some of traits analyzed, such as the acorn volume and mass, did indeed show strong variability (high coefficients of variation) among individuals within the same population, which obviously limits their reliability as discriminant traits among the genetic groups. However, traits such as the acorn height and width, cupule width, and peduncle length and diameter showed greater consistency and relatively low intrapopulation variability (Table S2); in fact, they were smaller than some leaf traits frequently used for species delimitation [20]. Although differences in the leaf morphology also allowed clear discrimination between the two studied species, the hybrids showed leaf traits in most cases that were similar to *Q. faginea* [20]. The hybrids also shared five of the estimated fruit traits with *Q. faginea*, but, in most of the remaining fruit traits, the hybrids exhibited intermediate values that were significantly different from both parents, and the differences were consistent in the different climatic zones. This suggests that the discriminating fruit traits are characteristic of the different genetic groups, regardless of the changes that they experience in response to changes in the environmental conditions, and that the genetic differences have a strong influence on the morphology of the acorns [32,33]. Our work, therefore, highlights the usefulness of fruit characteristics to distinguish oak species and their hybrids, at least for our study complex.

Regardless of the study area, *Q. pyrenaica* showed larger acorns (length, width, and volume) and wider and thicker cupules, covering a smaller proportion of the length of the fruit (shorter CH/AH ratio, Figure 3), as well as shorter and thicker peduncles, while the opposite was observed for *Q. faginea*. Previous studies have shown that large seeds tend to present increased germination rates in species such as *Quercus ilex* and *Castanea sativa* [34,35] and that larger seeds generally produce larger seedlings with higher relative growth rates, presumably because of the greater metabolic energy available for establishment, growth, and development [36,37,38]. Meanwhile, it has traditionally been proposed that larger acorns may suffer higher predation rates [34,39] for several reasons: because they take longer to develop, because optimal foraging theory suggests the predator’s preference for larger seeds over smaller ones, and because larger seeds may host both large and small insects [40,41,42]. However, in our study, *Q. pyrenaica* was the species with the fewest signs of consumption in all study areas, with the highest values recorded in *Q. faginea*, the genetic group with the smallest acorns. Therefore, other factors, apart from the acorn size, may also determine individual differences in predation. *Quercus* species are well known for their extreme interannual variability in fruit production (masting effect) [24]. The “predator satiation hypothesis” states that masting allows a greater proportion of acorns to escape predation by satiating predators during years of high acorn production and by reducing predator populations during years without acorn production [43,44]. Although both of our study species maintained a strong tendency for mast production, *Q. pyrenaica* is characterized by more marked and frequent interannual variations in acorn production [24]. *Q. pyrenaica* was the genetic group with the highest acorn production in the three zones across the gradient. In fact, the acorn production in *Q. pyrenaica* was, in all cases, much higher than the average, according to existing classifications [45]. On the other hand, larger and thicker cupules, such as those shown by *Q. pyrenaica*, may play an important role in protecting acorns. Besides providing physical protection, the high phenol levels in the cupules in many species of *Quercus* may prevent animals from feeding on the acorns prematurely [46,47].

Along the climatic gradient, we observed important differences in the three genetic groups, both in acorn production and in their characteristics. Given their influence on pollination, fertilization, ovulation, and flowering, it has traditionally been considered that the climatic conditions decisively contribute to determining acorn production [48,49]. In general, it tends to be considered that higher temperatures and lower water availability would result in lower levels of production, with water deficits being the main driver, rather than the temperature per se [21,50]. In our case, acorn production tended to be lower in the warm zone and, with slight differences, higher in the coldest area. However, drought seems unlikely as an explanation for these differences in production because, in our study, the warm zone registered the highest levels of precipitation, which, in principle, would allow it to compensate for its higher temperatures, resulting in similar levels of water stress between the zones. Furthermore, the differences between the cold and intermediate zones cannot be explained by precipitation, given the similar levels of annual rainfall between both areas. Similarly, several authors have reported no relationship between seed production and precipitation [22], and others have even been unable to find a significant relationship between acorn crops and the weather (precipitation and temperature) in different oak species [51,52]. In some cases, decreases in acorn production have been attributed to biotic factors, with herbivores affecting fecundity in oaks and causing significant losses of acorns due to infestation by bacterial pathogens that enter through holes or cracks caused by these herbivores [53,54]. In our opinion, however, the most probable explanation for the differences in acorn production among our sites is the trade-off between the acorn size and number, as pointed out by other authors [55,56], since the individual acorn mass tended to decrease with decreasing temperatures across the gradient (Figure 2).

The acorn traits showed clear plasticity in response to changes across the environmental gradient, with a greater width but smaller length and mass, as well as longer and thinner peduncles and smaller and generally thinner cupules, as the temperature decreased (Figure 2 and Figure 3). Negative intraspecific correlations between the size and/or mass of acorns and an increase in latitude or altitude, mediated by a decrease in temperature, have been observed in different *Quercus* species [57,58]. Several authors have proposed that the increase in the size and mass of acorns makes it possible to slow down water loss in warmer environments [59,60,61]. In fact, positive intraspecific correlations between the seed mass and xerothermic indices have been obtained in different *Quercus* species [62]. In the same sense, it could be interpreted that the larger and thicker cupules that we found in the warmer areas through the gradient play an important role in limiting evaporative losses when the fruit is on the tree [59,63]. However, in our case, differences in water availability were unlikely to be responsible for these trends in the fruit morphology across the gradient because, as mentioned above, the warm zone registered the highest levels of precipitation. Rather, we posit that these trends could be due to the unfavorable effects on the acorn size of lower primary productivity in colder environments at higher latitudes and altitudes, as other authors have pointed out [64]. In the coldest sites examined in our study, low temperatures and late frosts can extend well into the growing season, imposing a delay in leaf emergence [20], which limits the productivity when the water conditions are still favorable during spring, before the appearance of the summer drought typical of Mediterranean environments.In a previous work on the same species as in the present study [20], our results revealed that greater plasticity in leaf traits could confer an advantage to *Q. faginea* in coping with changing environmental conditions in the future. However, from the point of view of fruit traits, *Q. pyrenaica* seems to show more favorable traits, thanks to its trend towards mast acorn production, larger acorn sizes, and less predation, with *Q. faginea* at the opposite extreme. The predation results, however, must be interpreted with caution. Since acorn sampling was performed in autumn, we could not determine the number of acorns lost before sampling. Fruits may fall before maturity due to abortion, abnormal fruit development, or infestation [53]. In some cases, the losses occur at the early stages of fruit development, when the acorns are very small, and are difficult to quantify. There may have been interspecific differences in the number of premature losses that could not be determined with the data obtained in the present study. *Q. pyrenaica* also tended to exhibit stronger responses to environmental changes between the climatic zones, with the plasticity indices being higher in general than in *Q. faginea* and the hybrids for some of the fruit traits. Greater phenotypic plasticity has been considered important to allow plants to respond successfully to changing environmental conditions [26]. Therefore, our results suggest that the vegetative and reproductive traits (leaves versus fruits) vary independently between our three study groups (*Q. faginea*, *Q. pyrenaica*, and hybrids) and in response to climatic factors, as other authors have also observed in other *Quercus* species [65]. With respect to the hybrids’ traits, both the results of our previous study regarding the leaves [20] and the results of the present study regarding the fruits seem to corroborate the fact that hybrids tend to show intermediate traits between both parent species. Traditionally, the persistence of parental species in the absence of reproductive barriers has been explained by the lower fitness of the hybrids [66,67]. Our results, however, do not indicate that, with respect to the production and traits of their fruits, hybrids exhibit transgressive characteristics that could justify their lower competitive capacity with respect to their parental species. In any case, the results of the present study highlight the importance of including, along with the leaves, the analysis of the traits of the fruits to differentiate and characterize species and their hybrids and to help predict the distribution of the three groups in a future scenario.

In summary, fruit traits seem to have discriminant value in differentiating among the three genetic groups. At the contact zones, hybrids exhibit intermediate fruit traits, which may explain their persistence under intermediate environmental conditions. More research is needed to verify whether the results of the present study may be extrapolated to other Mediterranean areas and whether the same differences are present in other masting episodes.

## 4. Materials and Methods

### 4.1. Selection of Sites and Individuals and Sampling

This study included nine sites located in three areas covering the regions of Castilla-León and Extremadura (Central–Western Spain). The three zones, due to differences in latitude and altitude, could be classified as cold, middle, and warm, mainly in terms of the absolute minimum temperatures and number of frost days per year (Table S4). The warm zone had the highest values of precipitation (which helped to reduce the differences in the intensity of drought stress between the sites), with a negligible difference between the cold and intermediate zones. In each area, three sites were selected (with a distance of less than 50 km from each other and therefore with few climatic differences between them): one with the apparent dominance of *Q. faginea* (in the absence of *Q. pyrenaica*), another with *Q. pyrenaica* (in the absence of *Q. faginea*), and intermediate sites with the presence of both species. The climate data corresponded to the average of five study years (2020–2024), during which the air temperature was recorded (at 10-min intervals) through sensors with data loggers (Hobo Pendant temperature/light datalogger, Part UA -002-08, Onset Computer Corporation, Pocasset, MA, USA), with a range of −20° to 70 °C and accuracy ± 0.53 °C from 0° to 50 °C, placed for this purpose at each site (two temperature sensors per site). Precipitation data were provided by the Spanish National Meteorological Institute.

At each site, around 30 specimens (located at least 5 m apart) of each of the dominant groups (*Q. faginea*, *Q. pyrenaica*, or hybrids) were selected according to their leaf morphologies and duly identified and geolocated. All selected trees were fully sun-exposed, mature specimens with heights between 8 and 10 m and diameters (at 1.3 m) between 40 and 60 cm. Leaf samples were collected from each specimen throughout 2021, and the genetic structure analysis and categorization were performed using AFLPs, following the method implemented in Structure Harvester version 0.6.93 [68], through the specific procedure detailed in [20]. Hybrid existence was determined by analyzing the admixture coefficient (Q) values. Supported by the previous literature [69,70], a threshold of Q ≥ 0.9 was set to distinguish purebreds from hybrids. In accordance with the obtained Q values, finally, of the 272 specimens sampled, the genetic analyses assigned 70 individuals to pure *Q. faginea* (26%), 76 individuals to pure *Q. pyrenaica* (28%), and 126 individuals to hybrids (46%) (the final assignment of individuals to genetic clusters within each of the three study areas is shown in Table S5).

Once the individuals had been categorized, at each site, we selected 10 trees from the dominant genetic group and at least 30 mature and apparently undamaged acorns per tree were collected directly from the trees in autumn 2024. The acorns were taken in the middle part of the periphery of the crown and in southern exposure. Groups of acorns from the same tree were packaged together in plastic bags and immediately transported to the laboratory, where they were first immersed in water to visually identify insect-damaged or infected or dead acorns. The proportion of acorns infested by insect larvae was counted for each tree. Abnormal and defective acorns were then discarded before the remaining acorns were stored in hermetically sealed plastic boxes in the dark at 4 °C until morphological characterization. Furthermore, in the field, on every tree, acorn production was evaluated using the visual survey technique developed by Koenig et al. [45], consisting of two observers on opposite sides of the tree, counting all acorns seen in a 15 s period; the index of acorn production was the total number counted per 30 s of observer time. The counting methods used at all sites closely matched the acorn counts obtained using other methods, such as traps and production categories, offering a fast and efficient method of assessing fruit or cone crops [45,52,71].

### 4.2. Determination of Fruit Morphological Characteristics

Measurements of 15 characteristics, selected among the most used from the literature [17,65,72], were taken from a total of 900 acorns (100 per site, 10 randomly chosen per tree). These characteristics, with their definitions and units, are summarized in Table 1.

The fruits were separated from the cupules and the length and width of the fruit were measured with a digital caliper (Digimatic Micrometer, Mitutoyo, Tokyo, Japan) with accuracy of 0.01 mm. The acorn width (AW) refers to the distance between the widest points on the left and right sides of the fruit, and the acorn height (AH) refers to the distance from the bottom to the top of the fruit. The height-to-width ratio (AH/AW) of each fruit was then calculated. Each fruit was measured three times and the data were averaged. The individual acorn volume (AV) was approximated assuming a prolate spheroid shape, using the formula $AV=\pi \cdot AH\cdot {AW}^{2}/6$ [65]. The acorn fresh mass (AFM) and acorn dry mass (ADM) (after oven-drying for 17 h at 103 °C) were measured with an analytical balance (Sauter AR70, Sauter, Ebingen, Germany), and the acorn moisture content (AMC) was later estimated as (AFM−ADM)/AFM·100. Moreover, the cupule height (CH), width (CW), and thickness (CT) (difference between the inner and outer diameters of the cupule) were collected with the digital caliper and then the ratios between the cupule height and width (CW/CH) and between the cupule and acorn height (CH/AH) were estimated. Assuming that a cupule is a semi-ellipsoid rotating around its longitudinal axis, we calculated the cupule volume (CV) via the equation $CV=\pi \left[CH\cdot {CW}^{2}-(CH-CT){\left(CW-2CT\right)}^{2}\right]/6$ [65]. Finally, the peduncle length (PL) and diameter (PD) were measured with the digital caliper.

### 4.3. Data Analysis

A value for each trait analyzed was obtained for each tree as an average of the 10 acorns measured in each case; finally, a value for each trait, genetic group, and site was estimated as an average of the 10 trees sampled in each case. An analysis of variance (ANOVA) was carried out to search for differences among the genetic groups (two parental species and hybrids) and among the three study areas for the different parameters considered, followed by a post hoc Tukey–Kramer HSD test (at a significance level of *p* = 0.05). Prior to any analysis, data were tested for normality (using the Kolmogorov–Smirnov statistic test) and variance homogeneity (Levene’s test) to proceed with data transformation when necessary. Acorn counts for each tree were log-transformed [log (x + 1)] for analysis [45]. Similarly, in the analyses involving fractional data (percentage of acorns infested by insect larvae), the data were logit-transformed [ln (p/(1 − p))] [73]. The variability in the acorns characteristics within each species and zone was measured by calculating the coefficient of variation (CV). Furthermore, for the traits in which significant differences were detected between zones, a plasticity index (PI) ranging from 0 to 1 was calculated as (maximum value-minimum value)/maximum value [74,75]. This index was used to verify which genetic group (*Q. faginea*, *Q. pyrenaica*, or hybrids) and which traits showed greater responsiveness to environmental changes across the gradient. Principal component analysis (PCA) was used to detect the most differentiating morphological traits for acorns and to assess the contribution of environmental factors to the variability in acorn traits. As a first step, a Pearson correlation matrix was used to determine associations between the variables, with log transformation used when necessary. The SPSS v28 statistical package was used to analyze the data (SPSS Inc., Chicago, IL, USA).

## Figures and Tables

**Table 1:** List of acorn characteristics examined, definitions, and units.

Fruit Traits		
AH	Maximum acorn height	mm
AW	Maximum acorn width	mm
AH/AW	Acorn length to width ratio	dimensionless
AV	Acorn volume	mm^3^
AFM	Acorn fresh mass	g
ADM	Acorn dry mass	g
AMC	Acorn moisture content	%
**Cupule traits**		
CH	Cupule height	mm
CW	Cupule width	mm
CT	Cupule thickness	mm
CV	Cupule volume	mm^3^
CW/CH	Cupule width to height ratio	dimensionless
CH/AH	Cupule height to acorn height ratio	dimensionless
**Peduncle traits**		
PL	Length of the peduncle	mm
PD	Peduncle diameter	mm

**Table 2:** Eigenvalues, percentage of the variance explained by the first two principal components, and factor scores of acorn traits (abbreviations and units as in Table 1).

	PC1	PC2
Eigenvalue	8.21	4.11
Percentage variance	54.8	27.4
Cumulative percentage	54.8	82.2
**Fruit traits**		
AH	0.930	0.171
AW	0.680	−0.697
AH/AW	0.030	0.919
AV	0.827	−0.506
AFM	0.960	0.045
ADM	0.957	−0.088
AMC	−0.171	0.555
CH	0.510	0.780
CW	0.969	0.032
CT	0.728	0.385
CV	0.876	0.360
CW/CH	0.577	−0.729
CH/AH	−0.444	0.709
PL	−0.742	−0.432
PD	0.870	0.269

**Table 3:** Plasticity indices ((maximum value-minimum value)/maximum value) for the different acorn traits (abbreviations as in Table 1) in each genetic group.

Fruit Trait	*Q. pyrenaica*	*Hybrids*	*Q. faginea*
AH	0.22	0.18	0.20
AW	0.15	0.19	0.14
AH/AW	0.34	0.32	0.31
AV	0.09	0.18	0.07
AFM	0.30	0.35	0.47
ADM	0.22	0.29	0.43
AMC	0.15	0.10	0.06
CH	0.30	0.25	0.29
CW	0.21	0.13	0.19
CT	0.38	0.29	0.28
CV	0.63	0.61	0.57
CW/CH	0.12	0.14	0.14
CH/AH	0.13	0.11	0.12
PL	0.35	0.35	0.26
PD	0.34	0.29	0.27

## Data Availability

The authors will make the raw data supporting this article’s conclusions available upon request.

## Supplementary Materials

The following supporting information can be downloaded at: https://www.mdpi.com/article/10.3390/plants14050718/s1, Table S1: Results of ANOVA for the effects of genetic group and climatic zone on the different morphological acorn traits analyzed. Abbreviations and units as in Table 1; Table S2: Coefficients of variation for the different acorn traits within each genetic group and site. Abbreviations as in Table 1; Table S3: Pearson’s correlation coefficients for the different acorn traits measured using the average values obtained for each tree (abbreviations and units as in Table 1). *p* > 0.05 NS; 0.01 < *p* < 0.05 *; 0.001 < *p* < 0.01 **; Table S4: Characteristics of the study sites. Climatic data were obtained as an average of five years (2020–2024); Table S5: Individuals sorted by Q value for genetic clusters K1 (*Q. faginea*) and K2 (*Q. pyrenaica*).
